# Parfitian Priority, Gene Therapy, and Disability

**DOI:** 10.1093/jmp/jhag005

**Published:** 2026-03-14

**Authors:** Dominic Wilkinson

**Affiliations:** University of Oxford, Oxford, United Kingdom

**Keywords:** gene therapy, medical ethics, prioritarianism, utilitarianism

## Abstract

Gene therapies for severe genetic disease are often highly expensive. In deciding whether or when to provide them, one ethical consideration is the benefit of treatment relative to cost. Another and separate consideration is concern for medical need and the desire to benefit those who are worse off. The latter is a prioritarian concern. But how should we apply prioritarianism to decisions about gene therapy, particularly since such treatments might affect which individuals come into existence? That question is the main aim of this paper. I focus on a particular version of prioritarianism articulated and defended by Derek Parfit. My primary aim is to explore how, if we were to adopt such an account, we should interpret and practically apply this in medical ethics. In doing so, I assess how it fits with other elements of Parfit’s philosophy. I defend a new “time-relative” version of priority.

## I. INTRODUCTION

Advances in medical technology such as gene therapy highlight familiar questions about the allocation of resources. Many novel therapies are extremely expensive: even well-resourced healthcare systems must make difficult choices about what they provide. Policy makers are likely to consider the effectiveness of genetic treatment (its impact on quality and length of life) relative to its cost. Another ethical factor is the concern for how badly off patients are (their medical “need”). Patients with a particularly severe form of genetic illness may be thought to have a stronger claim to receive treatment. As with decisions about other scarce or expensive treatments, health systems might draw on both utilitarian and prioritarian considerations.^1^

The nature of genetic illness potentially raises some distinctive issues. Treatments may be provided for patients with a range of severity of illness at different time points (e.g., before or after symptoms develop, or even at the embryonic stage) and potentially in ways that affect which individuals come into existence (e.g., through embryo selection or embryonic/fetal gene therapy). Should we prioritize treatment for existing patients with severe symptoms?^2^ How should we evaluate this, compared with treating patients with milder illness, or compared with non-individual-affecting ways of preventing the illness? (Because my focus in this paper is largely on policy decisions about providing or funding gene therapy, I use “we” to refer to policymakers.)^3^

This question might lead to different types of ethical or philosophical enquiry. On a practical level, we might wish to know how the severity of genetic illness should factor into cost-effectiveness modelling and funding allocation decisions. At a more theoretical level, we could assess whether prioritarianism is correct or which version of the theory to apply. My focus in this paper is slightly different. I am interested in exploring a particular, influential account of ethical prioritarianism—that developed by Derek Parfit. My goal is to identify how Parfit’s account would or should be applied to practical questions relating to treatment allocation and gene therapy. The paper is partly exegetical—describing and clarifying Parfit’s view. However, I also suggest and defend extensions of the view that are consistent with Parfit’s overall approach.

To help anchor discussion (and because it exemplifies the dilemma), I start by describing a paradigm case of severe genetic disease (Spinal Muscular Atrophy [SMA]), and the options and effectiveness of gene therapy (including interventions that may affect which individuals come into existence). I then explore Parfit’s prioritarian account, identifying its key features and potential variants and how these relate to treatment allocation decisions. I argue that Parfit’s prioritarianism should be understood to be moderate and pluralistic. It should be applied in a way that is temporally neutral. I introduce the concept of time-relative priority as a way of connecting Parfit’s views on priority and identity. As I defend, one way to reconcile different elements of Parfit’s philosophy would be to give priority to those who are worse off at the time or within a period of time to which they are strongly psychologically connected. Finally, I suggest that on a Parfitian account, prioritarianism should be interpreted in a way that is reasons-agnostic and individual-affecting, albeit supplemented by impartial principles. I draw some conclusions about gene therapy.

### Case: Gene Therapy for Severe Disability

A newborn infant is found at birth to have contractures of his joints and marked weakness of his muscles. His breathing is weak, and he needs support with his breathing.

The infant is suspected to have a genetic problem and testing confirms that he has the most severe form of a rare disorder, “Spinal Muscular Atrophy.” Previous infants with this condition have all died early in infancy. Recently, however, gene therapy has emerged. This treatment appears to be effective at slowing progression of the disorder, but is extremely expensive. Current funding arrangements would pay for the treatment for less severely affected infants, but not in this case. Is this decision justified?

### Spinal Muscular Atrophy

Spinal muscular atrophy is a rare genetic condition that causes loss of nerve cells to the skeletal muscles (Wirth et al., 2020). Patients develop progressively worsening muscle weakness affecting movement and (in more severe cases) breathing.

The classic form of SMA (so-called “type 1”) affects infants who appear normal at birth but start to develop signs of muscle weakness in the first weeks or months and gradually lose virtually all strength (Wirth et al., 2020). Without treatment, they develop respiratory failure and usually die in the first year of life.

Less severe forms manifest in later infancy (type 2), childhood (type 3), or adulthood (type 4). The later-onset forms progress more slowly and lead to less severe disability and milder or no breathing involvement. There is also a rare, even-more-severe form that causes muscle weakness in utero and early neonatal death (type 0, see case above) (Matesanz et al., 2020).

Until relatively recently, all treatment for SMA was supportive. However, there are now a number of new therapies (Ramdas and Servais, 2020). This includes a gene therapy (onasemnogene abeparvovec, Zolgensma) that is often cited as the most expensive medication in the world—costing approximately US $2million per treatment (Strauss et al., 2022). It limits the progression of disease but does not tend to reverse established muscle weakness. There are other treatments currently in clinical use. To simplify discussion, I concentrate on Zolgensma.

#### Symptomatic treatment

Treatment can be applied after symptoms develop and a diagnosis is made. In the United Kingdom, Zolgensma is approved and funded for symptomatic treatment of most patients with SMA type 1, but not for either more severe, nor for less severe forms (National Institute for Health and Care Excellence, 2021). In one evaluation, Zolgensma was assessed to cost $243,000 per Quality-Adjusted Life-Year (QALY) gained for treatment of SMA type 1 (Dangouloff et al., 2021). Incremental cost-effectiveness for treatment of less severe forms in studies ranged from $400,000 to $8 million per QALY.

One reason for not offering treatment to the more severe forms is lack of evidence. There is anecdotal evidence (Matesanz et al., 2020) but limited data on cost-effectiveness (National Institute for Health and Care Excellence, 2021). This points to a general problem: patients with rare disease (or rare forms of disease) may be disadvantaged because of a lack of data to inform funding decisions. I largely set that epistemic consideration aside in what follows and assume for the sake of argument that there is at least some evidence of effectiveness in more severe forms of illness. The UK National Institute for Health and Care Excellence (NICE) (2021) concluded that treatment of more severely affected infants was likely to be substantially less cost-effective than treatment of SMA 1. This is because of greater muscle weakness before commencing treatment and higher supportive costs (Panagiotou, Kanaka-Gantenbein, and Kaditis, 2022).

#### Presymptomatic treatment

Treatment could be applied before symptoms developed—for example, where parents have had a previously affected child and testing is initiated in utero or at birth. Alternatively, newborn screening can identify infants with SMA who have not yet developed symptoms (Aragon-Gawinska et al., 2023). Such pre-symptomatic treatment is more effective and cost-effective than treatment after symptoms have developed. In the United Kingdom, pre-symptomatic treatment with Zolgensma is funded for infants <12 months with type 1, 2, or 3 SMA (National Institute for Health and Care Excellence, 2023).

#### Germline gene therapy and selection

While it has not yet been attempted, gene therapy could, in theory, be applied even earlier: for example, in cases where couples have undertaken IVF, have had pre-implantation genetic diagnosis, and have an affected embryo. Of course, another alternative in that instance would be selectively to implant unaffected embryos. (Germline gene therapy would be likely to involve different technology than that used postnatally. It may therefore involve different relative costs and effectiveness. I set aside such differences for this paper and concentrate on how we should evaluate it if it were equal in cost/efficacy.)

How should we evaluate these different options? One ethical theory has been suggested to have important insights.

## II. PRIORITARIANISM

The idea of giving ethical *priority* to those who are worse off is sometimes attributed to contractualist philosophers such as John Rawls, but one of the most significant proponents was the philosopher Derek Parfit. In his 1984 book, *Reasons and Persons*, Parfit (1984) defended what many have interpreted as an impartial, utilitarian approach to ethics. However, in an influential 1991 lecture and a follow-up paper, he explicitly analyzed and classified a set of ethical concerns about the importance of distributing benefits in a way that conflicted with utilitarianism (Parfit, 1991, 2012).^4^

Parfit considered examples like one suggested by Thomas Nagel (1979). In his book *Mortal Questions*, Nagel imagined parents who have two children, one who is happy and healthy and a second who has a painful disability. One parent is about to change jobs, and they are considering two options. If they move to the city, they could access specialist medical treatment for the second child, but housing is more expensive, and quality of life and opportunities would be worse for the first child. Alternatively, they could move to a semi-rural suburb. This would be better for the first child (in opportunities and quality of life, etc.), but worse for the second.

In his hypothetical example, Nagel makes it clear that the welfare gain for the first child in moving to the suburb is greater than the gain for the second child in moving to the city. If the parents were simply concerned about achieving the greatest good overall, they should move to the suburb. Yet, there is an intuitive concern that the needs of the disabled child are more pressing and that this should motivate the parents to favor them over the healthy child.

Nagel’s book chapter was titled “equality,” and he described this sort of concern as “egalitarian,” but Parfit distinguished two different reasons for making decisions that would favor the worse-off child. One reason is because this would make the outcome for the children more equal (the disabled child would end up closer in well-being to their healthy sibling; this is a genuinely egalitarian reason). But a different reason is out of concern for the worse-off child. The thought is that benefitting them matters *more*. Parfit labelled this “The Priority View.”

In cases like Nagel’s children, concern for equality and concern for the worst-off would yield the same answer. However, Parfit identified that these can also diverge. Imagine the following situation, inspired by the example at the start of the paper. Policy makers can afford a limited amount of gene therapy and are considering a group of children, half of whom have been identified with a severe form of SMA, and half with a milder form. They could

- Treat no children. 10 children will have severe SMA, 10 will have milder SMA.
- Treat half of each. 5 children will have severe SMA treated, 5 will have milder SMA treated. (5 of each are untreated).
- Prioritize those who are worse off. Treat 10 children with severe SMA (10 children with milder SMA are untreated).
- Genetic disenhancement. Give 10 children with milder SMA a gene treatment that makes them more severely affected—equivalent to the severe form (20 children have the equivalent of severe SMA).

### Egalitarian Gene Therapy Allocation

If we are concerned about inequality, each of these options might be attractive, though in different ways. Options A and B treat children with severe and milder forms of genetic illness the same way (in one case by treating neither of them)—but in both cases there is substantial natural inequality that remains. Option C involves treating the children unequally but improves the outcome for those who are worse affected (and hence, reduces overall inequality). Option D makes the mildly affected children worse off but generates a more equal outcome. (In another way, this outcome is more unequal. There are now a greater number of severely affected children, who are worse off than non-affected children. A different way of achieving a more equal outcome would be to give *non*-affected children genetic disenhancement so that they all have the equivalent of mild SMA.)

Although option D (or disenhancement of non-affected children) is to most people immediately unattractive, Parfit argued that cases like this provide a powerful counter-example to some forms of egalitarianism. In many situations, one accessible way to reduce inequality is to make some people (those who have higher well-being) less well-off. While many egalitarians would recognize that in Option D the improvement in equality is potentially outweighed by the harm of making some children much worse off, Parfit (1991) claimed that it is *in no way better* to make some people worse off but benefit no one. This is what he called the *Levelling Down Objection*. An ethical view that suggests that this is some kind of improvement is, he argued, simply mistaken. In place of egalitarianism, Parfit proposed a Priority view that placed special importance on benefits to the worse off. Importantly, this view appears to yield the right answer to cases like Nagel’s children, without being susceptible to the leveling-down objection.

There are two key attributes to Parfit’s Priority view that distinguish it from egalitarianism. The first is that although such a view, by giving priority to the worse off, tends to reduce inequality over time, it gives no intrinsic value to equal distributions. Second, the priority to the worse off is not relative to the well-being of others. Our special reason to give treatment to children with severe SMA in option C above would be no less if there were in fact no children with milder SMA; nor would it be any greater if there were 100 children with milder disease. When we give priority to the worse off, that is not because they are *worse than other people*. Rather, it is because they are worse off relative to some absolute standard.

Last, it is worth correcting a common misconception. On the Priority view, the lower an individual’s well-being (the worse off they are), the stronger the reason to benefit them. However, it is important to distinguish that from a different claim. This is the claim that we can *most benefit* those who are worse off.

When we are allocating resources (whether those are medical or financial or otherwise), there is often diminishing marginal utility. If we allocate to those who are already in good health, or well-off, etc., we make a smaller difference than if we allocate the same resource to those in poor health, etc. (Gene therapy will have a smaller impact on well-being for those with type 4 SMA than for those with type 1.) Diminishing marginal utility often leads utilitarians to favor allocation to those who are worse off—since that would lead to the greatest benefit.

But that is different from the Priority view. On the priority view, we have a stronger reason to provide *the same* benefit or even a smaller benefit to those who are worse off beforehand. For example, if we use Quality Adjusted Life Years (QALYs) as our way of quantifying the benefit, it is not that treatment will yield more QALYs if directed to those who are worse off. Rather, the claim is that the same (or fewer) QALYs are *more important* to provide.

When we have such a stronger reason to beneﬁt one of two people, we can say that, even if this beneﬁt would be in itself *smaller*, it would have greater moral weight, or be a greater weighted beneﬁt. (Parfit, 2012, 42).

## III. INTERPRETING PARFITIAN PRIORITARIANISM

Parfit’s seminal paper distinguished prioritarianism from egalitarianism. However, his description explicitly or implicitly included a number of variants of his new theory. This includes those listed in table 1.

**Table 1.** Theoretical variants of Prioritarianism^a^

Weight of priority	Strong (absolute)	Moderate
Temporal bias	Present	Neutral
Unit of priority	Lifetime	Time-specific
Nature	Telic	Deontic
Application to non-identity cases	No (individual affecting)	Yes (impartial/non-individual affecting)
Other values	Pure priority	Priority pluralism

a This suggests that there are 32 theoretical versions of prioritarianism, with different combinations of these views, albeit some combinations are unlikely. Nils Holtug has noted other potential variations relating to the scope of prioritarianism: geographic (whole world or just domestic) and species (just human beings, or also non-human animals) and its application in the setting of uncertainty (Holtug, 2017).

To understand the implications of Parfit’s view for cases like Gene Therapy, we are going to need to decide which form of the Priority View we should apply.

### Moderate

One worry about prioritarianism is that it might lead us to devote very large amounts of healthcare resources to those who are extremely badly off, but whom we have little, if any ability to help. For example, we might devote all of our treatment resources to patients with severe (type 0) SMA, even if the effectiveness of treatment were very limited. Such a conclusion clearly arises from some forms of prioritarianism (including Rawls’ difference principle) (Parfit, 1991). But the unattractiveness of this implication might lead some to reject prioritarianism, or alternatively, to adopt a more moderate form.

Parfit was inclined to a moderate view. He wrote that on his Priority View: “benefits to the worse off *could* be morally outweighed by sufficient benefits to the better off [emphasis added]” (1991, 20). On such a moderate view, we might choose to treat patients with SMA type 1 (infantile) rather than the more severely affected type 0 because the significantly greater benefit of treatment in the former group outweighs the higher priority in the latter group.

However, this same conclusion might be justified solely on the basis of utility and cost-effectiveness. If we wished to give ethical weight to priority, health systems should potentially use priority weighting or use a higher cost-effectiveness threshold for those who are very badly off (see, e.g., Adler, 2011; Ottersen, Mæstad, and Norheim, 2014; Reckers-Droog, van Exel, and Brouwer, 2019) That would mean that even if gene therapy for SMA type 0 would not ordinarily be thought to be sufficiently cost-effective, nevertheless, it may be provided. Various countries, including the United Kingdom, give extra weight in their deliberation to severity of illness (Ottersen et al., 2016; Wailoo, 2024).

Parfit’s prioritarianism is clearly “moderate.” However, this leaves two challenges. First, Theron Pummer (2016) has argued that even moderate forms of prioritarianism are theoretically vulnerable to a form of reductio ad absurdum—a “Priority Monster.” Pummer contends (drawing analogies with Robert Nozick’s Utility Monster and with Repugnant Conclusion-style arguments) that this implies that we should give miniscule, barely perceptible benefits to someone extremely badly off rather than large (significant) benefits to another badly off person. Responses to this argument could include either a finite limit to the priority given to worse-off individuals or a finite limit in the real world to how badly off someone can be.^5^

Second, if like Parfit we are drawn to a moderate view, the challenge remains of deciding how much weight to give to priority for the worst-off versus utility. Parfit acknowledged that there was no clear way to determine this, apart from relying on judgment. He referred to this as an “intuitionist” view, since it requires judgment in balancing the relevant principles (Parfit, 1991, 5).

### Temporally Neutral

One of the ways of providing gene therapy for SMA is to give it before symptoms develop. For example, newborn infants can be identified through screening tests performed after birth. For SMA, there is good reason to think that such pre-symptomatic treatment is *more effective* than treatment after symptoms develop. That is because the genetic defect leads to progressive loss of spinal motor neurons. Even if the genetic defect is corrected, those neurons do not regenerate. Treatment after symptoms manifest may halt progression of weakness but does not typically restore completely normal function.

From the point of view of utility and cost-effectiveness, health systems might prefer pre-symptomatic treatment to symptomatic treatment. But would the Priority view suggest the opposite? After all, it appears that patients who have symptoms (muscle weakness, breathing problems) are worse off than those who do not (yet)?

In his description of the Priority view, Parfit did not clearly address this question. Nevertheless, it is clear from some of his other writing that he would have rejected such a conclusion. There are relevant differences between present and future harms (e.g., relating to predictability and uncertainty, and arising from our weakened psychological connection with further future selves) (Parfit, 1984). However, in general, Parfit strongly defended a temporally neutral approach. The timing of when a harm occurs is not, he argued, relevant to the badness of that harm (Parfit, 1984). The same conclusion applies to the timing of priority.

If we are temporally neutral in our application of the Priority view, then that implies that we should consider prevention and treatment of severe illness symmetrically. In situations where (as is the case for SMA) prevention is more effective, that implies a clear preference for funding the former.

It is worth highlighting here an ostensible, contingent advantage of prevention over treatment (Wilkinson, 2023). In a number of cases of severe illness, the effectiveness of treatment diminishes with increasing severity (particularly beyond a certain point). This applies to SMA, but it also applies to, for example, treatment of strokes, traumatic head injury, or heart attacks. In such cases, priority of treatment conflicts with utility since priority would favor those most severely affected, while utility can go in the opposite direction.^6^ But that does not apply to prevention. In many instances (and again, this applies to SMA), preventative interventions are equally effective for very severe and mild illness. Where that applies, priority and utility both favor prevention of more severe illness. The old saw that “an ounce of prevention is worth a pound of cure” is justified both because prevention is overall more effective but also because it is ethically less conflicted.

I have argued that the timing of treatment is not relevant. It follows that (other things being equal) the timing of prevention is also ethically not relevant. Accordingly, prevention by embryonic gene therapy would have equivalent priority to postnatal gene therapy. Is that right? I return below to priority of interventions that change who comes into existence. Some have argued that gene therapy at the embryonic stage is non-individually affecting—that is, that it would change the identity of the subsequent children brought to birth (Zohar, 1991; Sparrow, 2022).^7^ In a separate paper, I have explored some of the challenges of determining whether such cases are individual-affecting or not and, hence, the difficulty of applying so-called two-tier views (Wilkinson, 2024). For this paper, I assume that if such cases are non-individual affecting, we should treat them with the same approach that we do IVF and embryo selection. If they are individual-affecting, we should treat them equivalently to post-natal prevention. But there is an additional complication.

### Time-Relative

There is a different debate within prioritarianism about when we identify someone as being worse off. Parfit noted that we could prioritize those who are worse off at the time (Temporal or Time-Specific Priority) or we could give priority to those who are worse in their lives as a whole (Lifetime Priority) (Parfit, 1991).^8^ These two views can diverge.

In his writings on prioritarianism, Parfit did not explicitly favor one of these accounts over the other.^9^ However, Parfit’s earlier writing was much more clearly skeptical of the idea of taking into account well-being over a lifetime. His view on personal identity led him to reject the idea of a single fixed individual over a lifetime. Instead of a single self, we may have a series of successive selves. If that is correct, it creates a problem for taking into account well-being across different points in a life. If we give someone priority for treatment because (in their early life) they previously had reduced well-being, on Parfit’s account that is analogous to giving someone priority for treatment because *another* person in the past had low well-being. He wrote:

only the deep further fact [an all or nothing, determinate fact about identity] makes possible compensation over different parts of a life. Since there is no such fact, we ought, as Nagel suggests, to change “the size of the units over which a distributive principle operates.” (Parfit, 1984, 346)^10^

Immediately after this, Parfit implies that these units should shrink to “people’s states at particular times” (i.e., adopt Time-Specific Priority). But there is a more modest possibility that Parfit did not explicitly contemplate and that has not previously been identified. We could adopt:

*Time-relative priority*: We should give priority to those who are worse off at the time, or within a period of time to which they are strongly psychologically connected.^11^

This would make the application of prioritarianism consistent with Parfit’s view about what matters from an individual’s point of view. An individual’s claim to benefit would plausibly be stronger if they are currently, or have recently been, badly off. Their claim would be correspondingly weaker if they had attenuated connections with the earlier self who was badly off.

How would this be evaluated? One approach to priority weighting identifies severity of illness as the difference between the well-being of a patient (or group of patients) and that of average members of the population. In the United Kingdom, this is calculated for future well-being, and yields an absolute or proportional deficit in Quality-adjusted life-years (the greater the deficit, the higher the priority) (Wailoo, 2024). The Norwegian approach is to calculate the expected health loss over a patient’s whole lifetime (Ottersen et al., 2016). Time-relative priority would yield a different answer. It would look at how badly off someone is but would discount both future and past well-being decrements in proportion to the prudential unity relations between the current and future/past self. With a high degree of discounting, this will approximate Time-Specific priority, while a lower degree of discounting would yield answers closer to lifetime priority. It is beyond the scope of this paper to identify in detail how this form of the priority view should be applied, but here are some suggestions. Time-relative discounting would potentially be symmetric for future/past well-being (because it is temporally neutral) but would also potentially be non-exponential. It may resemble the sort of hyperbolic discount that is evident in public preferences (but which is often characterized as irrational) (Ahmed, 2020). It is also perhaps important to note that this Parfitian argument for discounting applies to priority weighting (i.e., in identifying who is worst off) but does not apply to actual well-being increments from treatment (i.e., the calculation of the QALY gains).

There are other practical reasons for favoring time-relative priority. One reason is that in many circumstances it is much more accessible to identify someone’s current (or recent) level of well-being. Particularly for older patients, it would be challenging to assess and compare how well-off/badly off they have been over their life to that point. Furthermore, “lifetime” priority also potentially asks us to weigh up future well-being—factoring in how long a life someone would have, how much well-being they will have in the far future, but such predictions are often difficult or uncertain.

Time-relative priority would also avoid another potential pitfall of lifetime priority. The latter would give priority to those with the lowest amount of well-being over their life. In theory, then, very short lives would have the highest priority—including genetic illnesses that lead to fetal or early embryonic loss. But that conflicts with the general intuition that it is much more morally important to address the health problems of children or adults than of early embryos (Ord, 2008).

There are different ways of responding to this latter concern. For example, we may believe that our life does not begin until the onset of the capacity for consciousness or self-consciousness—that would exclude priority for embryos. Alternatively, it may be that the greatest priority is for those with the most *negative* well-being. Those who die very early in life may not have much positive well-being, but they probably also do not have large amounts of negative well-being. But the time-relative view also has a plausible answer. It would identify the badness of death as linked to both the amount of future well-being lost and the strength of the psychological connections with that future well-being (McMahan, 2002). On this view, it is worse to die as a five-year-old child, than to die as an infant, than to die as a fetus. Correspondingly, even though the latter would result in shorter lives, their treatment would have lower time-relative priority than the five-year-old with a life-threatening illness.

The implications of this Prioritarian account for the Gene Therapy case at the start of the paper depend on how we evaluate the interests of newborn infants relative to older infants or children. Infants with type 0 (congenital) SMA are worse off in one way than infants who develop symptoms at a later stage (e.g., 6 weeks, 3 months, 18 months, etc.). But the older infants may be worse off in another way, because they have stronger time-relative interests in continued life (Wilkinson, 2013), and then we need to balance these two effects.

### Reasons-Agnostic

There is a more fundamental (though perhaps more abstract) question that could be asked about ­prioritarianism. What is the nature of the moral reason to give priority to the worse off? Parfit (1991) identified two different forms of the Priority view:

*The Telic Priority/Prioritarian view:* Benefiting people **makes the outcome better**, the worse off the people are who receive those benefits.

*The Deontic Priority view*: We have **a stronger moral reason** to benefit people, the worse off the people who receive those benefits are (though that would not make the outcome better).

There are different ways of defending the latter, but one that Parfit described is

*Claim Prioritarianism:* People have stronger moral claims to receive some benefit the worse off these people are. (Parfit, 2012, 437)

Should we be telic or deontic prioritarians? In his lecture Parfit wrote, “for most of my discussion, this difference does not matter” (1991, 20). This apparent agnosticism is consistent with Parfit’s (2011) later work in which he defended the idea that plausible forms of consequentialism, deontology, and contractualism would converge (he used the analogy of different theories climbing a mountain from different sides).

If we follow Parfit’s arguments here, the conclusion might be taken to be a negative one—there is no need to decide which theory is correct to decide practical questions about the application of prioritarianism. However, it may be useful in a different way. We could see competing theories as providing useful moral triangulation. It might be easier to reach a conclusion from some directions rather than others, just as sometimes the path up a mountain is more easily travelled on one side rather than another. Agnosticism may also sometimes clarify. If we cannot justify an element of prioritarianism from one perspective (e.g., deontic), that may mean that we need to reject that element. I return to that possibility.

### Individual Affecting

I noted that one way of preventing SMA is through IVF and embryo selection. Such an approach would be considerably cheaper than gene therapy and potentially more effective (depending on when gene therapy was administered). However, it also would not *benefit* the children who would have been born with SMA. A different child or children would be born instead. (This is a “non-individual affecting” case) (McMahan, 2009).

How should we understand non-individual-affecting interventions on the Priority view? One possibility is that the view does not apply. At the end of his 2012 paper, Parfit wrote:

… [T]he Prioritarian Principles that I have considered cannot be applied to cases in which, in the different possible outcomes, different people would exist. When we consider these cases, we need other principles. (2012, 440)

Parfit was specifically referring here to decisions to have children, and rejecting the idea that there are prioritarian reasons to bring someone into existence. On the priority view, benefits matter more, the worse off someone is (absent that benefit). But a non-existent individual is not badly off if they are not conceived: they do not exist. Shlomi Segall has suggested that this implies a form of “Quarantined Prioritarianism,” that is, that prioritarianism should *not* be applied to cases of a particular type (Segall, 2022). But there are two types of non-individual-affecting case. The first are those that affect the size of future populations (“different number cases”). As Parfit noted, it appears problematic to apply the priority view to these.^12^ However, the second type of case is those, like our gene therapy versus embryo selection example, that do not affect population size (“same number cases”).^13^

In his final book, *On What Matters*, Parfit explicitly considered same number of cases in his discussion of the non-identity problem and Scanlon’s contractualist formula. There he argued that standard contractualist accounts ought to be supplemented by “impartial reasons” (Parfit, 2011). These are reasons that we all have, regardless of our personal point of view. For example: “We would all have strong impartial reasons to want there to be a thousand people who would live for 80 years, rather than a thousand different people who would live for only 40 years” (Parfit, 2011, 240).

One way of understanding these impartial reasons, suggested by Shlomi Segall (2022), is that existential benefits^14^ (the benefit of bringing individuals into existence) are weighted so that benefits at lower absolute levels count for more. On this explicitly telic account, non-individual-affecting interventions (like embryo selection) would potentially be evaluated in a similar way to individual-affecting ones. That would favor more equal well-being in future populations.

However, although that solves one problem, it conflicts with several of the arguments that I outlined above. First, this appears to conflict with Parfit’s notion of ethical convergence. It is not clear that we can explain non-individual-affecting cases in deontic or contractualist ways.^15^ For example, it cannot be that a possible individual who would be brought into existence at low levels of well-being (e.g., with SMA) has a “claim” that a different individual with higher well-being should exist instead of them.^16^

Second, this idea of comparing the well-being of one possible child with a different possible child conflicts with Parfit’s central idea that prioritarianism is about absolute rather than relative well-being. Recall that when we give priority to the worse off, that is supposed to be independent of the well-being of other people.

The third challenge is that it is unclear how to consider a *time-relative* form of priority when making decisions about who will come into existence. At which time point would we be applying priority, if we are considering whether to select an embryo without an SMA mutation, rather than one with the mutation? How would we or could we discount well-being decrements for someone who does not exist? A lifetime priority account would be easier to apply, though as I argued above, such an account is hard to reconcile with Parfit’s other views.

Where does this leave us? The majority of cases of prioritization in health care (including decisions about resource allocation and funding of gene therapy) involve comparison of benefits to individuals and consideration of their level of well-being. For those individual-affecting cases, the Priority view provides a clear reason for giving priority to the worse off.

There are also ethical considerations that apply in non-individual affecting cases—impartial reasons that cannot easily take a deontic or contractualist form. This might lead us to conclude that Priority view is inherently individual-affecting (i.e., it cannot be applied to cases like embryo selection). At the very least, the considerations that apply in non-individual-affecting cases seem to have a *different* nature from standard Prioritarian reasoning.

One important question is how we should weigh up the reasons that we have in individual-affecting and non-individual-affecting cases, are they equally strong, or do they differ? Do we have a greater moral reason to (for example) provide a gene therapy to a child with SMA than to select an unaffected embryo? Parfit (2011) strongly rejected the idea that only individual-affecting reasons matter. That then leaves two possibilities: that there is no difference between non-individual-affecting and individual-affecting reasons (the “no-difference view”) or that they have different moral weight (a “two-tier view”). I have elsewhere defended the view that in some cases there can be a stronger (deontic) reason to bring about an individual-affecting benefit (e.g., applying to parents), though this does not apply to policy decisions about funding treatment (Wilkinson, 2024). That seems to imply that from a policy perspective we should consider embryo selection in a similar way to postnatal treatment (i.e., in terms of its costs and benefits).

### Pluralistic

Finally, we should consider whether prioritarianism provides a sufficient basis for decisions about allocating treatments like gene therapy, or whether it needs to be supplemented and balanced by other principles.

In some of his writing, Parfit seemed inclined to the former. He suggested that an advantage of the Priority view (over egalitarianism) was that it “can be held as a complete moral view” (Parfit, 1991, 22). Yet in other places, he acknowledged that prioritarians may not use the theory in this way.

When I introduced the Priority view … [I] claimed that versions of this view had been held by various earlier writers, all of whom are moral pluralists, who accept other principles. (Parfit, 2012, 429)

Of course, in one way, moderate prioritarianism is intrinsically pluralist in that it involves balancing the value of utility with priority for worse off. Furthermore, in the preceding section, I identified that prioritarian principles appear to need supplementing with impartial principles for non-individual affecting cases. Next, in reply to a criticism of prioritarianism, Parfit defended his view partly by alluding to other principles. He was responding to a claim that in some cases individuals are morally required (on Prioritarian grounds) to make decisions about their own future well-being that they would not rationally prefer (because they would be expectably worse). However, Parfit claimed that:

If Prioritarians accept a pluralist moral view, of the kind that Sidgwick calls common sense morality, their view has no such implausible implication. (2012, 424)

The pluralism question points to a central tension in Parfit’s philosophy. Like his role model Sidgwick, Parfit was strongly motivated to seek overarching theories that could resolve disagreement and provide ethical progress. However, he also (like Sidgwick) ultimately recognized the different sources of normativity and the need to balance these.

The above brief arguments suggest that even if the Priority view could theoretically be held monistically, it need not be. Furthermore, it appears most plausible to include it as one element of a wider moral framework. That would undermine the claimed “complete theory” advantage over egalitarianism, though other advantages would remain.

## IV. CONCLUSION: GENE THERAPY AND PARFITIAN PRIORITARIANISM

I have argued that Parfit’s prioritarianism should be understood and applied in a way that is moderate and pluralistic, temporally neutral (though time-relative), agnostic (about the source of normativity), and individually affecting (though potentially supplemented by impartial principles). How does that help us overall with the questions with which we started about prioritizing treatment for severe genetic disease?

Before summarizing some potential conclusions, it is important to acknowledge the limited scope of the foregoing analysis. I have endeavored to clarify one prioritarian account. But Parfit is not the only prioritarian, and other writers would reach different conclusions about some of these questions.^17^ Furthermore, I have not, directly, sought to defend Parfit or prioritarianism against their critics.^18^ If I am correct in my conclusions about how to interpret this ethical theory, it does not follow without further argument that this is the theory we should adopt.

However, notwithstanding these reservations, this analysis may be useful in part because (as in the case at the start of the paper), questions about whether, when, or how we should give priority to the worse off commonly arise in medical ethics. Parfit’s prioritarian account remains highly influential, though he was sometimes ambiguous about which form it should take. Finally, philosophical discussions about prioritarianism are sometimes hard to connect to real-world debates about treatment and severity of illness. It is important to understand what implications should be drawn from them, and how the conclusions would overlap with or deviate from current practice.

I have argued, first, for the intuitively plausible (and obvious) idea that prevention is better than cure. From the point of view of prioritarianism, we should usually prefer preventive interventions (e.g., treating patients pre-symptomatically) because they have equivalent *priority*, but often greater utility than treating symptomatic patients. This provides support for screening programs for genetic disease—for example, through whole genome newborn screening or potentially through prenatal diagnosis.

Next, where symptoms have developed, we have priority-based reasons to give extra weight to the severity of manifestations (including how badly off a patient is and has recently been), though this must be balanced against utility. I have articulated and defended a novel version of the Priority view that is consistent with Parfit’s view on what matters prudentially. Priority for a life-threatening illness would potentially alter with the individual’s time-relative interests in continuing life (yielding lower priority to fetal or early neonatal illness). For SMA, that may not change the current policy of funding of treatment for type 1, but not funding treatment for the most severe type 0. However, on a prioritarian account, some extra weight for severity should be included in cost-effectiveness analysis.

For families who have been identified as carriers of SMA, they might choose IVF and embryo selection (or prenatal diagnosis and termination of pregnancy). Such an intervention would be *considerably* less costly than embryonic or later gene therapy, though the benefit would be non-individual affecting. There are important but distinct questions about how we should respond to individual-affecting versus non-individual-affecting ways of preventing future disability. Elsewhere, I have argued that in terms of funding policy, we should treat them symmetrically (Wilkinson, 2024). In that case, selection or prenatal testing would be (because of lower cost) clearly preferable to embryo gene therapy.

Last, I have pointed out that if we include prioritarianism in decision-making, there is a need to balance different ethical considerations. That includes, obviously, priority versus utility, but it also includes individual versus non-individual-affecting considerations and other relevant ethical values, such as the weight given to the autonomy of parents. Since the theory itself does not provide any answer to the correct balance, we might need to engage with the public in a process of deliberation about these different factors and how to incorporate them into policy (Savulescu, Gyngell, and Kahane, 2021).

The emerging availability of therapies to treat and prevent severe genetic disorders offers considerable benefit but also brings with it difficult choices. Priority for those who are or will be very badly off is not the only ethical factor to take into account. But it is one, potentially highly relevant factor. We need to be clear about how to apply it in ethical decision-making.

## Footnotes

1 As an example of nongenetic illness, during the COVID pandemic there were questions about whether patient frailty should be used for triaging patients for admission to intensive care (Wilkinson, 2020). More severely frail patients were predicted to have a higher mortality from COVID (and so might have a lower chance of benefiting from intensive care admission) but were also potentially worse off.

2 Later I distinguish between telic and deontic forms of prioritarianism. As explored elsewhere (Wilkinson, 2024), the duties of parents may be significantly different from those of public health officials.

3 Other authors who have explored the broader argument for applying prioritarianism to gene therapy include Farrelly (2004).

4 There are several extant versions of the first lecture. A shortened version with a slightly different title “Equality *and* Priority” [emphasis added] was published in *Ratio* in 1997 (Parfit, 1997).

5 There may be maximum amounts of negative well-being that someone could experience. I return below to the significance of very short lives.

6 In other cases, utility can increase with severity—because of a greater difference in well-being between those treated and untreated, even if those with severe illness have incomplete resolution of symptoms.

7 This argument might extend to postnatal gene therapy. If the subsequent phenotypic change were radical enough, this might change numerical identity on some views (Elliot, 1993).

8 In his lecture, Parfit also identified two other possibilities. We might include both Temporal *and* Life-Time priority (Parfit called this “Double Prioritarianism”). He also referred to a different form of priority—according to urgency of need, which he largely set aside (Parfit, 1991).

9 He does imply that some objections to the Prioritarian view (by Otsuka and others) do not apply to Temporal or Dual Prioritarians (Parfit, 2012).

10 Parfit is here citing Nagel’s chapter on equality (Nagel, 1979). In fact, Parfit seemed to accept that it is plausible to reject this argument: “This conclusion is defensible, but so is its denial” (Parfit, 1984, 346), though it is difficult to see how such denials would be compatible with his views on identity.

11 This draws on McMahan’s concept of Time-Relative Interests (McMahan, 2002).

12 Separate from the conceptual worry, a different concern is that application of the priority view to such cases would lead to the “repugnant conclusion,” that is, a moral imperative to aim for a very large future population of barely happy people. Michael Otsuka (2022) suggests that this was Parfit’s main concern.

13
Segall (2022) argues that prioritarianism need not be (and should not be) quarantined from same number of cases (He also thinks that it can be applied in different number cases.

14 This term comes from McMahan rather than Segall.

15 For related reasons, Michael Otsuka (2022) claims that Parfit’s Priority view should be understood to be individual-affecting.

16 Nor can the possible individual with higher well-being (e.g., without SMA) have a claim to be brought into existence.

17 Others who have defended forms of prioritarianism include (Adler, 2011; O’Neill, 2012; Williams, 2012; McKerlie, 2013; Holtug, 2017; Arneson, 2022).

18 For a detailed defense of prioritarianism against criticisms, see Adler and Holtug (2019).
